# Thyroid Storm and Diabetic Ketoacidosis Presenting to the Emergency Department

**DOI:** 10.7759/cureus.6751

**Published:** 2020-01-23

**Authors:** Sean Griffiths, David Peak, Rachel E Bridwell, Brit Long

**Affiliations:** 1 Emergency Medicine, San Antonio Uniformed Services Health Education Consortium/Brooke Army Medical Center, San Antonio, USA; 2 Emergency Medicine, Brooke Army Medical Center, Fort Sam Houston, USA

**Keywords:** diabetic ketoacidosis, thyroid storm, thyrotoxicosis, diabetes mellitus

## Abstract

This paper diabetic ketoacidosis (DKA). Previous literature describes these two disease states presenting together, but most reports describe the etiology as uncontrolled diabetes leading to thyroid storm. After literary review, we present what we believe to be the first case of thyroid storm and DKA occurring in the same person on multiple occasions with an insulin pump. Additionally, we provide evidence that points to the thyroid storm being caused by DKA versus the traditional teaching of DKA precipitating thyroid storm.

## Introduction

Thyroid storm, a severe form of thyrotoxicosis, is an acute life-threatening complication of hyperthyroidism, which if left untreated may lead to irreversible cardiovascular collapse and death. The mortality rate for this condition is described as high as 10%-30% [1]. Diabetic ketoacidosis (DKA) is also a life-threatening situation where severe insulin shortage or resistance leads to anion gap metabolic acidosis, ketosis, hyperglycemia, and electrolyte imbalances [2]. Thyroid storm and DKA are both acute, potentially fatal complications of their respective pre-existing conditions; however, the coinciding development of both conditions is uncommon and may ultimately lead to death. Thus, early recognition and aggressive treatment are crucial in preventing further decompensation.

## Case presentation

A 28-year-old female with a history of Graves’ disease and type 1 diabetes mellitus presented to the emergency department complaining of one day of abdominal pain, nausea, and vomiting. Her past medical history included type 1 diabetes mellitus controlled by an insulin pump, Graves’ disease controlled by propylthiouracil, hypertension controlled with multiple medications, and Alport syndrome. She had recent difficulty controlling her blood glucose levels over the last several days and was unable to take her thyroid medications due to nausea and vomiting.

Upon arrival to the emergency department, she presented in obvious distress with a heart rate of 143 beats per minute (bpm), a temperature of 36.9 degrees Celsius, a blood pressure of 155/76 mmHg, a respiratory rate of 22 breaths per minute, and oxygen saturation of 99% on room air. Her clinical examination revealed a mildly confused, moderately obese patient, repeatedly vomiting, and complaining of abdominal and chest pain. Her lungs were clear when auscultated. Her abdomen was soft and moderately tender in the mid-epigastric region. Her skin was warm and diaphoretic, and she had bilateral lower extremity edema. Electrocardiogram showed sinus tachycardia with a rate of 150 bpm, and her chest radiograph was normal. During the exam, the patient endorsed a previous episode of DKA with concomitant thyroid storm, presenting in a similar fashion. A finger-stick blood glucose was 736 mg/dL. Her initial laboratory data revealed a lactate of 6.0 mmol/L, a potassium of 4.3 mmol/L, metabolic acidosis with an anion gap of 26.3, pH of 7.3, a free thyroxine level of 7.77 ng/dL, a suppressed thyrotropin (thyroid-stimulating hormone) level of less than 0.005 μIU/mL, and a ketonuria of 40 mg/dL. She was given a score of 50 according to the Burch-Wartofsky diagnostic criteria of thyroid storm-scores of > 45 points are highly suggestive of thyroid storm (Table 1) [3,4].

**Table 1:** Burch-Wartofsky Score

Burch-Wartofsky Scoring Table	Point System
Temperature (F)	
99-99.9	5
100-100.9	10
101-101.9	15
102-102.9	20
103-103.9	25
>104.0	30
Central nervous system effects	
Mild (agitation)	10
Moderate (delirium, psychosis, lethargy)	20
Severe (seizure, coma)	30
Gastrointestinal-hepatic dysfunction	
Moderate (diarrhea, nausea, vomiting, abdominal pain)	10
Severe (unexplained jaundice)	20
Cardiovascular dysfunction (heart rate)	
99-109	5
110-119	10
120-129	15
130-139	20
>140	25
Atrial fibrillation	10
Heart failure	
Mild (pedal edema)	5
Moderate (bibasilar rales)	10
Severe (pulmonary edema)	15
Precipitant history	
Positive	0
Negative	10
Total score	
<25	Thyroid storm unlikely
25-45	Impending storm
>45	Thyroid storm

The patient was treated with removal of the insulin pump, intravenous rehydration using two liters of lactated ringers, intravenous insulin drip started at 0.1 U/kg/hour, 4 mg of intravenous zofran, and 10 mEq of intravenous potassium chloride. To address her thyroid storm, the patient initially received oral propranolol 80 mg, oral propylthiouracil 400 mg, and intravenous hydrocortisone 100 mg. Medical intensive care and endocrine services were consulted, and she was admitted to the Medical Intensive Care Unit. During the patient’s hospitalization her anion gap and tachycardia resolved, she was switched to subcutaneous insulin, and on hospital day 3, she was discharged on a methimazole taper.

## Discussion

Thyroid storm and DKA represent two separate, but equally life-threatening conditions that require both early recognition and prompt treatment to prevent severe decompensation. Thyroid storm can occur in untreated hyperthyroidism but is most commonly attributed to an acute stressor, such as infection, burn, or metabolic derangement [4]. DKA is becoming an increasingly more common disease process with a reported mortality of >5% in elderly populations [2]. It remains the most common cause of death in pediatric and adolescent patients with insulin-dependent diabetes mellitus [2]. The combination of diabetes mellitus and DKA has been well established. However, the pathophysiology under which DKA and thyroid storm present together is still debated, and exact rate of simultaneous occurrence is currently unknown [5]. Although DKA is a known precipitant of thyroid storm, it can be easily appreciated how the converse, thyroid storm leading to increased insulin resistance, can lead to severe DKA [4]. Thyrotoxicosis alone alters carbohydrate metabolism and drives insulin resistance due to increased hepatic glycogen breakdown, while unsuppressed hepatic glucose production further ramps up the metabolic derangements [6]. Thyroid storm leading to DKA is especially plausible in the above case as the patient was unable to maintain her blood glucose levels despite escalating appropriate doses of insulin administered from her pump.

Previous literature has explored the relationship between thyroid storm and DKA, but to date no case reports have been published in a patient with an insulin pump. This leads to an important question of whether DKA or thyroid storm was the consequence or the precipitating factor [4,6-10]. One case report diagnosed DKA and thyroid storm in a patient without previous history of diabetes mellitus or thyroid disease [7]. Another published in 2016 described DKA and thyroid storm presenting as acute psychosis [8]. A systematic review of 21 cases of concomitant DKA and thyroid storm published in 2019 examined current literature and found many risk factors associated with both conditions, but it was unable to definitively link cause and effect of both conditions [5]. No case in this review contained a patient with an insulin pump but noted that drug noncompliance was a significant contributing factor in nearly all reported cases. Because our patient was noncompliant with thyroid medications due to PO intolerance, but maintained her insulin pump, it is likely thyroid storm precipitated her DKA, in contrast to previous examinations of thyroid storm that cite DKA as the common precipitating factor. Additionally, unlike the above patient, there were no case reports found that described DKA and thyroid storm occurring in the same patient multiple times.

The authors were fortunate that this patient was a reliable historian and had presented to our hospital previously; thus, her Graves' disease and diabetes mellitus were taken into account during her evaluation. This patient’s presentation and triage vital signs indicated severe disease, though the initial presentation can be significantly more subtle in elderly patients or those unable to mount tachycardia secondary to beta-blockers [9]. Previous case reports have shown that one of the principle features of thyroid storm, fever, can be masked when both disease processes present together, making recognition even more difficult [10]. 

## Conclusions

Thyroid storm and DKA are two life-threatening conditions that present to the emergency department, but their rare appearance together presents diagnostic uncertainty for clinicians requiring a high index of suspicion to recognize. Current literature does not quantify the frequency of this simultaneous entity. Emergency clinicians should consider these two disease states presenting together in patients with no known precipitants of their thyroid storm or DKA, as it is unclear which process leads to exacerbation of the other.
